# Diseases of Women

**Published:** 1894-01

**Authors:** 


Diseases of Women. By Henry J. Garrigues, A. M., M. D. Profusely illustrated. Price, cloth, $4.00, net; sheep, $5.00, net. In press. Ready shortly. W. B. Saunders, 925 Walnut Street, Philadelphia, Pa.

